# Dysbiosis of the gut microbiome in elderly patients with hepatocellular carcinoma

**DOI:** 10.1038/s41598-023-34765-w

**Published:** 2023-05-13

**Authors:** Weizheng Zhang, Xiaosong Xu, Liping Cai, Xiangsheng Cai

**Affiliations:** 1 Clinical Laboratory, Guangzhou Cadre Health Management Center, Guangzhou No. 11 People’s Hospital, Guangzhou, China; 2 Clinical Laboratory, The First Affiliated Hospital of Guangdong Pharmaceutical University, Guangzhou, China; 3 Department of Basic Nursing, Guangdong Province Chaozhou Health School, Chaozhou, China; 4 Institute of Translational Medicine, University of Chinese Academy of Sciences-Shenzhen Hospital, Shenzhen, China

**Keywords:** Microbiology, Oncology

## Abstract

Fecal samples from participants aged 60–80 were collected and sequenced by a high-throughput second-generation sequencer to explore the structural composition of gut microbiota in elderly patients with hepatocellular carcinoma(HCC). Comparison of gut microbiota between patients with hepatocellular carcinoma and healthy controls, α diversity and β diversity were statistically different. At the genus level, compared with the normal group, the abundance of *A Blautia, Fusicatenibacter, Anaerostipes, Lachnospiraceae_ND3007_group, CAG-56, Eggerthella, Lachnospiraceae_FCS020_group and Olsenella* were decreased significantly in the LC group. In contrast, the abundance of *Escherichia-Shigella, Fusobacterium, Megasphaera, Veillonella, Tyzzerella_4, Prevotella_2 and Cronobacter* increased significantly. The KEGG and COG pathway analyses showed that the dysbiosis of gut bacteria in primary liver carcinoma is associated with several pathways, including amino acid metabolism, replication and repair, nucleotide metabolism, cell motility, cell growth and death, and transcription. Age is negatively associated with the abundance of *Bifidobacterium*. *Lachnospiraceae_ ND3007_ group*, *[Eubacterium]_hallii_group*, *Blautia*, *Fuscatenibacter* and *Anaerostipes* are negatively correlated with ALT, AST and GGT levels (p < 0.05), respectively. Alpha-fetoprotein (AFP) is positively associated with the abundance of *Erysipelatoclostridium*, *Magasphaera*, *Prevotella 2*, *Escherichia-Shigella*, *Streptococcus* and *[Eubacterium]_eligens_group* (p < 0.05), respectively. A random forest model showed that the genera *Eggerthella*, *Anaerostipes,* and *Lachnospiraceae_ ND3007_ group* demonstrated the best predictive capacity. The area under the Receiver Operating Characteristic Curve of *Eggerthella*, *Anaerostipes* and *Lachnospiraceae_ ND3007_ group* are 0.791, 0.766 and 0.730, respectively. These data are derived from the first known gut microbiome study in elderly patients with hepatocellular carcinoma. Potentially, specific microbiota can be used as a characteristic index for screening, diagnosis, and prognosis of gut microbiota changes in elderly patients with hepatocellular carcinoma and even as a therapeutic clinical target.

## Introduction

Liver cancer is a common malignant tumor in clinical settings, with primary liver carcinoma being one of the most common malignant tumors in China^1^. Liver carcinoma still ranks among the top three causes of death of common tumors, which seriously threatens patients' health, life, and well-being^2^. The diagnosis of liver cancer is based on imaging, pathology, and molecular biology.

In terms of treatment and traditional local treatment, surgical resection and liver transplantation, more and more treatment schemes are implemented in clinics, including the treatment strategy with gut microbiota as the target^3,4^. The relationship between gut microbiota and primary liver carcinoma and the pathogenesis of related diseases has attracted extensive attention in recent years. Studies have shown that compared with the healthy control group, the *Firmicutes* in the feces of patients with liver cancer are significantly reduced, and the ratio of *Firmicutes* to *Bacteroidetes* is significantly reduced^5^. Additionally, *Pseudomonas*, *Candida albicans,* and *Staphylococcus* rate*s* in the intestinal tract of patients with liver cancer are higher than those of healthy controls. The occurrence of liver cancer may be related to the disorder of gut microbiota^6^, or some specific microbiota can be used as important targets for treating the disease^7^. The "gut-liver axis" theory holds that the intestine and liver are interconnected in anatomy and physiology. Intervening with the gut microbiota structure has become a new strategy for liver cancer prevention and treatment^8^.

However, there are many complex types of gut microbiota, and primary liver carcinoma pathogenic factors are also variable^9^. The correlation between gut microbiota and primary liver carcinoma is still unclear. Therefore, studying the gut microbiota characteristics and their correlation with primary liver carcinoma is practical and clinically relevant. It is well-known that elders have an increased risk of developing chronic diseases, including some cancers^10,11^. A study showed that 73% of the patients diagnosed with liver cancer are older than 65 years, and more than 43% of the patients are older than 75 years^12^. HCC occurring at younger and older ages have been thought to have distinct oncogenic mechanisms^13^ and possibly subsequent clinical courses^14^. The current clinical research on the correlation between hepatocellular carcinoma and gut microbiota does not consider the issue of age. The age range of hepatocellular carcinoma patients is extensive, and the gut microbiota varies substantially over a large age range. Therefore, this study focuses on gut microbiota variability in elderly patients with hepatocellular carcinoma.

## Materials and methods

### Sample data

Twenty-five patients aged 60–80 with hepatocellular carcinoma were included in the disease group. HCC was diagnosed either pathologically or radiologically according to guidelines for diagnosing and treating hepatocellular carcinoma in China–two of the four imaging studies, including computed tomography (CT), magnetic resonance imaging (MRI), gadoliniumethoxybenzyl-diethylenetriamine pentaacetic acid MRI, or contrast-enhanced ultrasonography, showing an arterial enhanced mass less than 2 cm, or one of the four imaging studies above showing an arterial enhanced mass greater than 2 cm^15^. Exclusion criteria include liver failure, liver transplantation, sepsis, renal failure, acute or chronic gastrointestinal diseases, autoimmune disorders, other uncontrolled life-threatening diseases, and recently consumed drugs or probiotics that affect gut microbiota structure (such as antibiotics, probiotics, and prebiotics).

Twenty-one healthy subjects aged 60–80 met the following criteria: (1) no history of liver disease; (2) normal blood/urine/stool routine tests and liver-kidney functions; and (3) no intestinal probiotics and antibiotics prescriptions within two weeks before sample collection.

Demographic details such as gender, age, and body mass index were comparable among the groups (Table 1). Each participant signed written informed consent before inclusion in this study. All experiments were approved and carried out following the guidelines of the Ethics Committee of Guangzhou Cadre Health Management Center(approval number: K2022-25). All methods were performed following the relevant guidelines and regulations.

**Table1:** Descriptive data of subjects.

	Control (n = 21)	LC (n = 25)	p values
Age (year)	68.57 ± 7.29	69.52 ± 7.32	0.6748
Gender			
Female	10	11	0.8061
Male	11	14	
BMI	23.57 ± 2.60	23.36 ± 3.70	0.8230
AFP (ng/mL)			
≤ 20	21(100%)	14(56%)	0.0005
> 20	0(0%)	11(44%)	
ALT (U/L)	16.6 ± 7.0	43.1 ± 34.2	0.00038
AST (U/L)	13.6 ± 5.3	53.7 ± 41.5	0.00002
GGT (U/L)	20.6 ± 7.2	84.7 ± 84.2	0.00036
DBil (μmol/L)	3.2 ± 0.9	13.0 ± 29.1	0.0663

### DNA extraction

Fresh fecal samples were collected from the participants on the day of the medical examination and immediately frozen at − 80 °C. Microbial DNA was extracted using a Tiangen fecal genomic DNA extraction kit according to the manufacturer's instructions.

### DNA amplification, library construction and microbiome data analysis

The V3V4 variable region of the bacterial 16S rRNA gene was used for amplification using polymerase chain reaction (PCR) with primers 806R (GGACTACHVGGGTATCTAAT) and 341F (CCTACGGGNGGCWGCAG). The resulting amplicons were then purified, pooled in equimolar amounts, and paired-end sequenced on Illumina HiSeq/MiniSeq for online sequencing. The raw data are processed using the BIPES protocol.10 and QIIME 1.9^16,17^. The sequence with 97% consistency is clustered into operational taxonomic units (OTUs) to obtain the abundance of each sample.

The indexes of observed species, Shannon, Chao1, ACE, J and Simpson, were used to calculate alpha diversity metrics. Principal component analysis (PCA) and principal coordinates analysis (PCoA) were analyzed using the unweighted UniFrac metric. The statistical significance of differences between groups was evaluated using analysis of similarities (ANOSIM). The Cluster of Orthologous Groups (COG) databases and Kyoto Encyclopedia of Genes and Genomes (KEGG) were used to analyze the pathway richness using PICRUSt^18,19^.

### Statistical analyses

Statistical tests were performed using Prism software (Graph Prism 7.0 Sofware Inc. CA, USA) and R 3.0.3 (R Foundation for Statistical Computing). The measurement data is represented by mean ± standard deviation (x ± SD). Wilcoxon's rank-sum test compared the diversities between any two groups. Fisher's exact test analyzed the categorical variables. Values of P < 0.05 were considered statistically significant. The random-forest classification was performed using the R package "random forest" to discriminate the samples from different groups. The model was employed for five-fold cross-validation of the relative species abundance profile. The performance of RF with the selected features was assessed using the receiver operating characteristic (ROC) curve and quantified by the Area under ROC (AUC)**.**

## Results

### Baseline data

Forty-six fecal samples from 25 HCC patients and 21 healthy subjects were collected and analyzed using 16S rRNA sequencing. Baseline participant information, including age, gender, and BMI, was collected. The clinicopathological information is presented in Table 1, showing that four groups had similar baseline characteristics regarding age, gender and BMI as revealed by variance analysis (p > 0.05). Liver function biomarkers, the levels of alanine aminotransferase (ALT), aspartate aminotransferase (AST), and gamma-glutamyl transferase (GGT) are significantly higher in HCC patients compared to healthy individuals (p < 0.05). There is no difference in direct bilirubin (DBil) between the two groups (p > 0.05).

### α diversity and β diversity analysis

**Figure 1:**
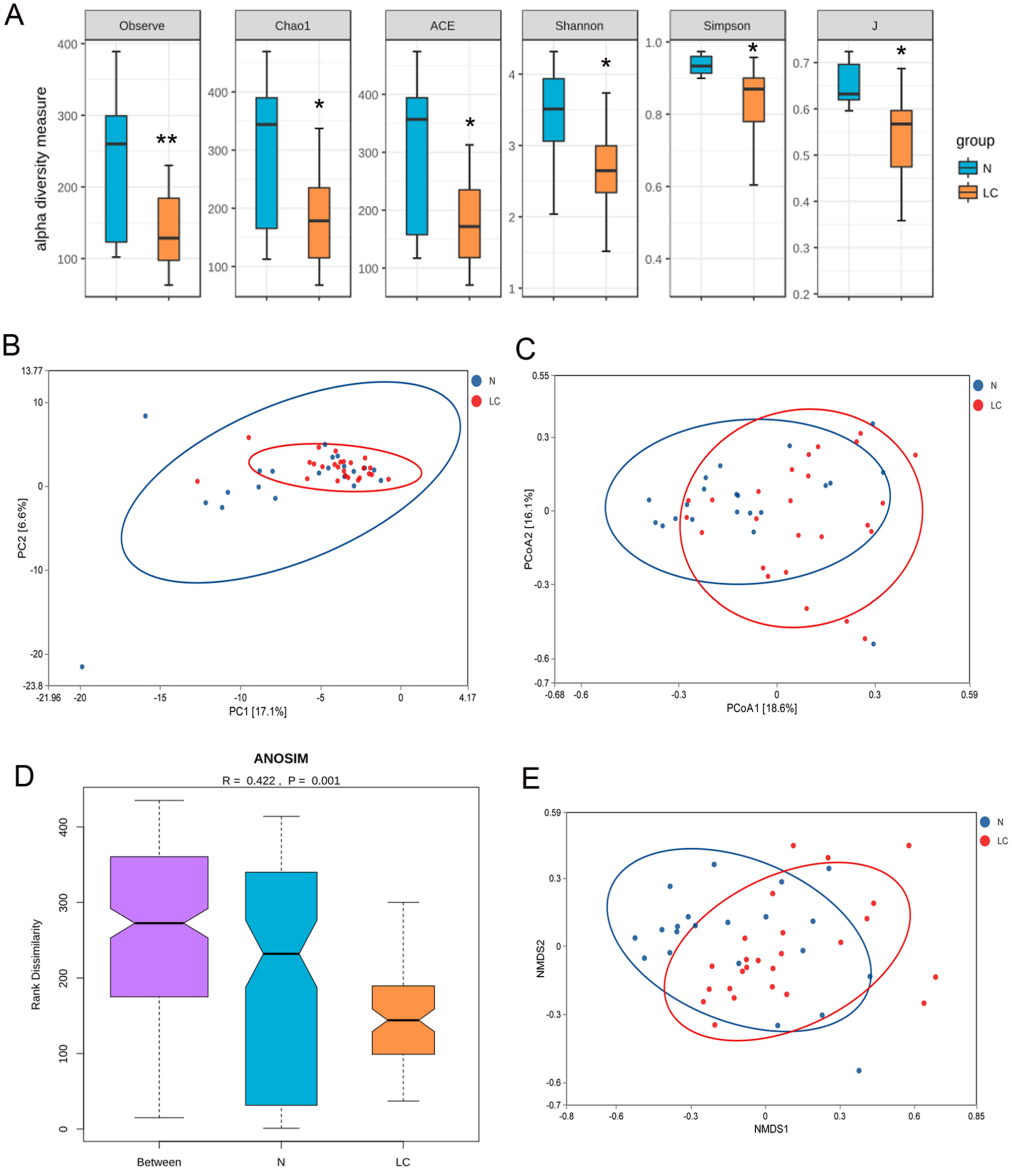
Gut microbiota alpha and beta diversity indices in elderly patients with HCC. (**A**) Gut microbiota alpha diversity in elderly patients with HCC. The Observed_species, Chao1, ACE, Shannon, Simpson and J values are shown,*p < 0.05, **p < 0.01. (**B**) PCA score plot based on the relative abundance of OTUs (97% similarity levels). (**C**) PCoA analysis. (**D**) Analysis of similarities. (**E**) Non-metric multidimensional scaling. Each dot represents a sample; the corresponding group can be found in the legend.

As shown in Fig. 1A, the results indicate that microbiota richness indexes (Observed_species, Chao1, ACE) in the healthy control group are significantly higher than in the disease group (p < 0.05). The Shannon, Simpson, and J indexes of species diversity in the control group are also higher than in the disease group (p < 0.05).

Results in Fig. 1B intuitively demonstrate that the OTUs of the control and disease groups are relatively aggregated in their respective groups. The contribution rate of the disease factor as the first principal component (PC1) to the microbiota difference is 18%. In comparison, the contribution rate of the difference between the samples as the second principal component (PC2) to the microbiota difference is 7%.

Additionally, based on the analysis of species composition differences, PCoA analysis was used further to explain the differences between the control and disease groups. As shown in Fig. 1C, the contribution rate of species composition difference between samples in the group as the first principal component (PCoA) to the microbiota difference is 18.9%. The contribution rate of species composition difference between samples in the group as the second principal component (PCoA2) to the microbiota difference is 12.5%. The comprehensive PCA and PCoA analysis data highlight noticeable differences between control and disease groups in microbiota species. Analysis of similarities (ANOSIM) indicates that the gut microbiota structure differs significantly between the LC group and healthy group (ANOSIM, r = 0.422, p = 0.001) (Fig. 1D). NMDS analysis reveals significant differences between LC patients and healthy individuals (Fig. 1E).

### OTU distributions

**Figure 2:**
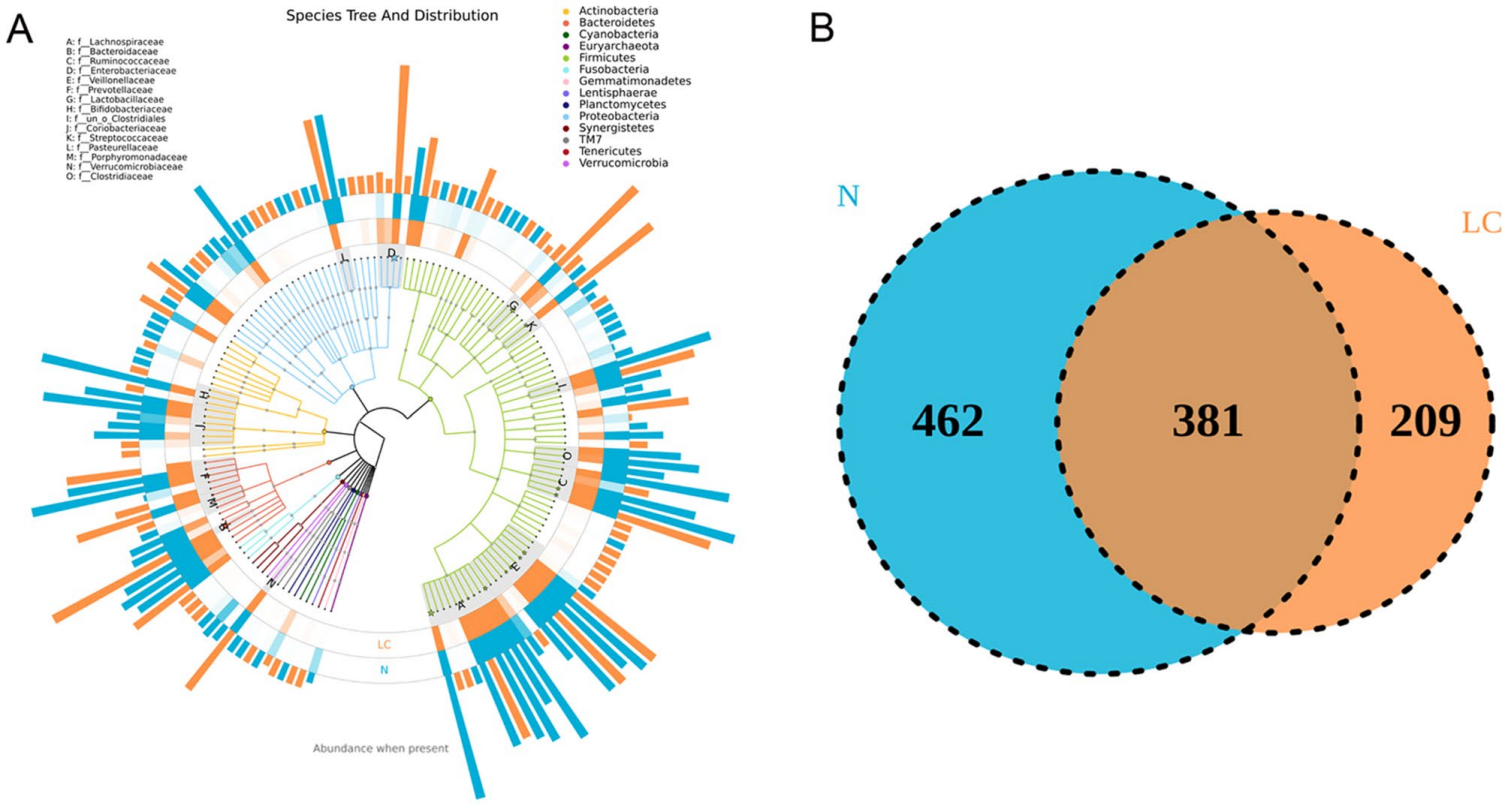
Operational taxonomic unit distributions. (**A**) Species tree and distribution of the gut microbial community. (**B**) Venn diagram showing the common or specific OTUs between the groups.

The microbiota-relative taxon abundance is compared with that in healthy subjects to explore the characteristics of the gut microbial community in LC patients. Sixteen hundred and four operational taxonomic units are annotated, including 22 phyla, 116 families, and 217 genera of gut microbes with 97% similarity among the samples (Fig. 2A). The Venn diagram in Fig. 2B reflects differences between the two groups and depicts 549 and 844 OTUs in the LC and control groups, respectively. Three hundred and eighty-one common OTUs are shared by the LC and control groups.

### Taxonomy

**Figure 3:**
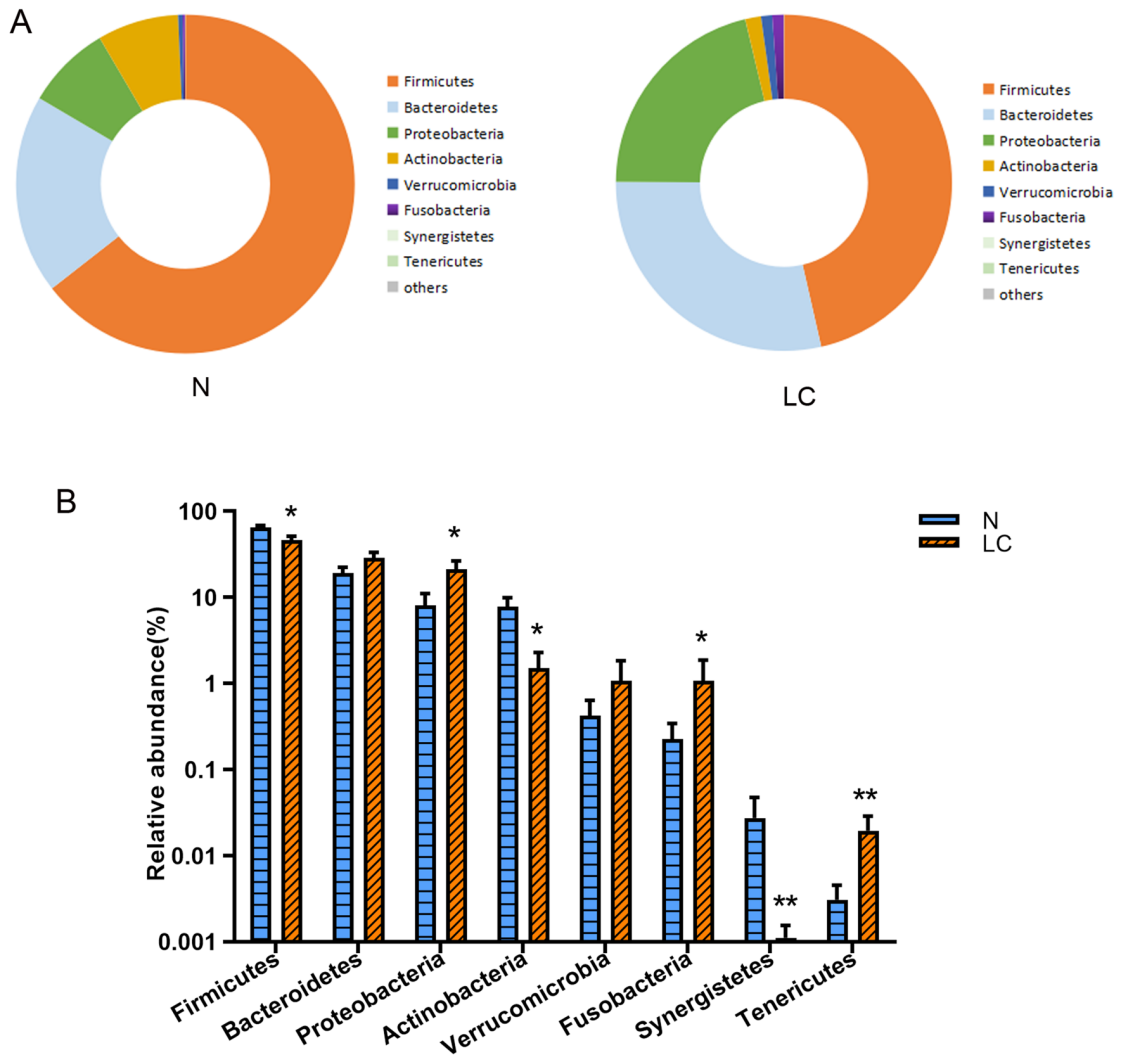
Taxonomic profile. (**A**) The OTUs were assigned to eight phyla, *Bacteroidetes*, *Firmicutes*, *Proteobacteria*, *Fusobacteria*, *Verrucomicrobia*, *Actinobacteria*, *Tenericutes* and *Synergistetes*. (**B**) Different bacteria were compared between each group at the phylum level. *p < 0.05, **p < 0.01, LC group compared with N group.

Twenty two phyla are annotated in this study, as shown in Fig. 3, in the taxonomic composition of the normal and disease groups. The abundances of *Firmicutes*, *Actinobacteria,* and *Synergites* in the liver carcinoma group are significantly lower than in the normal group (p < 0.05). The abundances of *Proteobacteria*, *Fusobacteria,* and *Tenericutes* increased significantly (p < 0.05). These six dominant bacteria account for the vast majority of all detected species (up to 99.9%).

**Figure 4:**
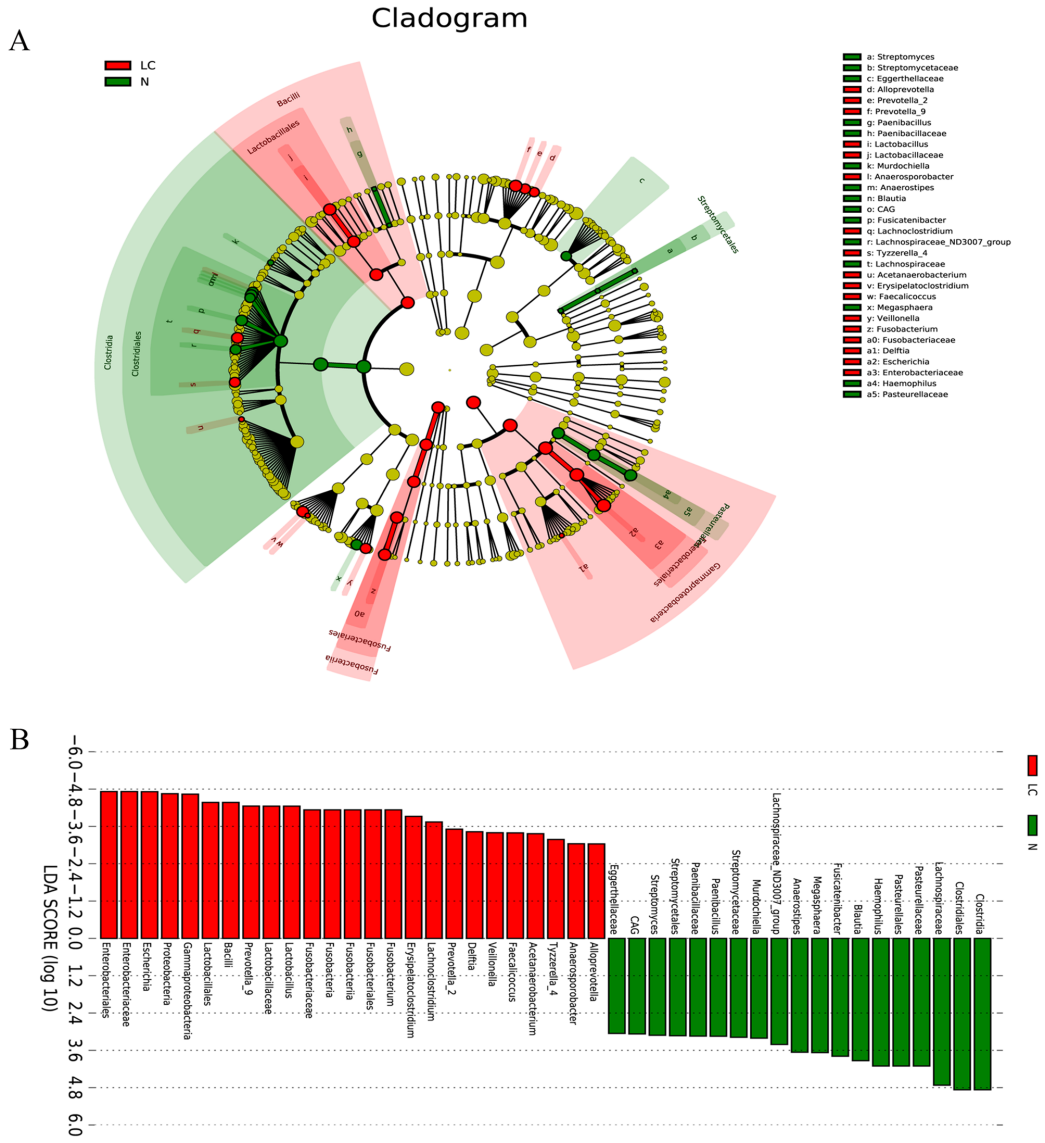
LEfSE analysis determined which bacterial taxa differed significantly between the groups. (**A**) The phylogenetic tree revealed the different enrichment taxonomies between N and LC groups. (**B**) Different taxonomy is shown with a bar plot.

Linear discriminant analysis effect size (LEfSe) analysis was performed to determine which bacterial taxa differed significantly between the groups. The phylogenetic tree revealed the different enrichment taxonomies between the N and LC groups (Fig. 4A). Different taxonomy was further extracted and shown with a bar plot (Fig. 4B).

**Figure 5:**
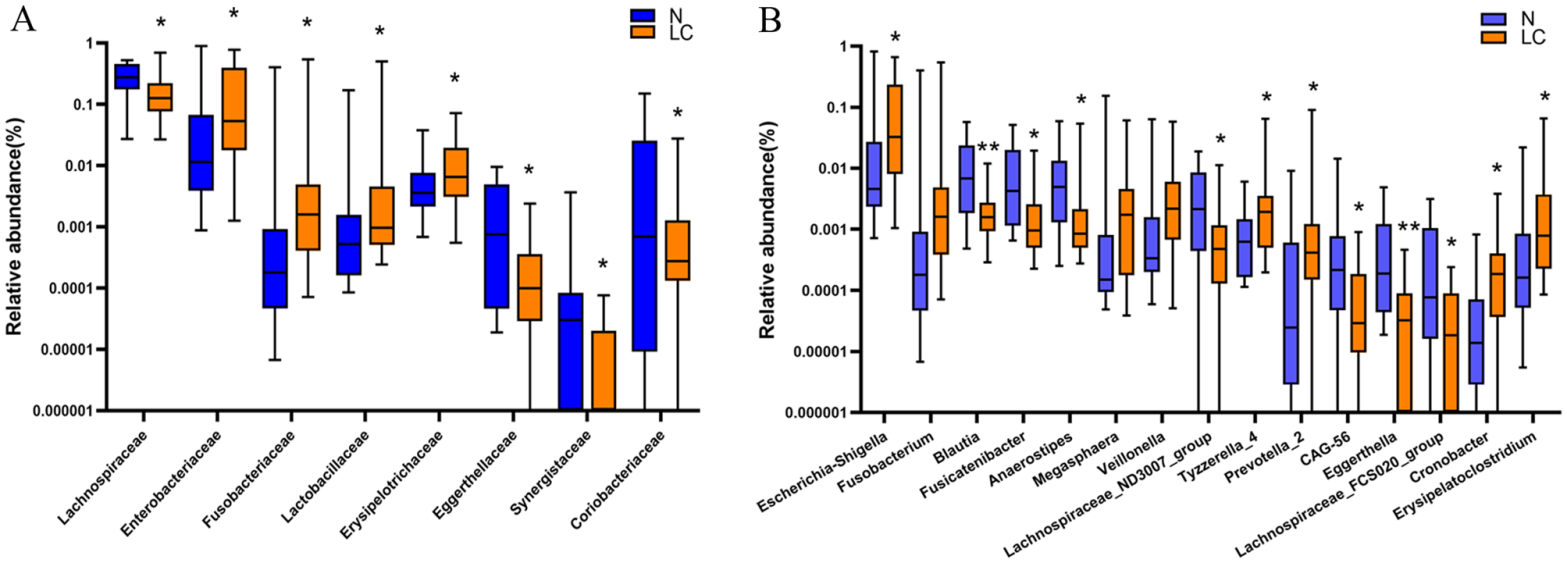
Composition of gut microbiota at the family (**A**) and genus level (**B**). *p < 0.05, **p < 0.01, LC group compared with N group.

The composition of the two groups of gut microbiota at the family level is shown in Fig. 5A. Compared with the normal group, the abundances of *Lachnospiraceae*, *Coriobacteriaceae*, *Eggerthellaceae* and *Synergistaceae* in the liver carcinoma group are decreased significantly (p < 0.05). The abundance of *Enterobacteriaceae*, *Fusobacteriaceae*, *Lactobacillaceae* and *Erysipelotrichaceae* increased significantly. The gut microbiota composition at the genus level is shown in Fig. 5B. Compared with the normal group, the abundances of *Blautia*, *Fusicatenibacter*, *Anaerostipes*, *Lachnospiraceae_ND3007_group, CAG-56*, *Eggerthella*, *Lachnospiraceae_FCS020_group* and *Olsenella* in the liver carcinoma group are decreased significantly (p < 0.05). The abundances of *Escherichia-Shigella, Prevotella_2, Tyzzerella_4, Cronobacter* and *Erysipelatoclostridium* are increased significantly (p < 0.05).

### Functional overview of the intestinal microbiome

**Figure 6:**
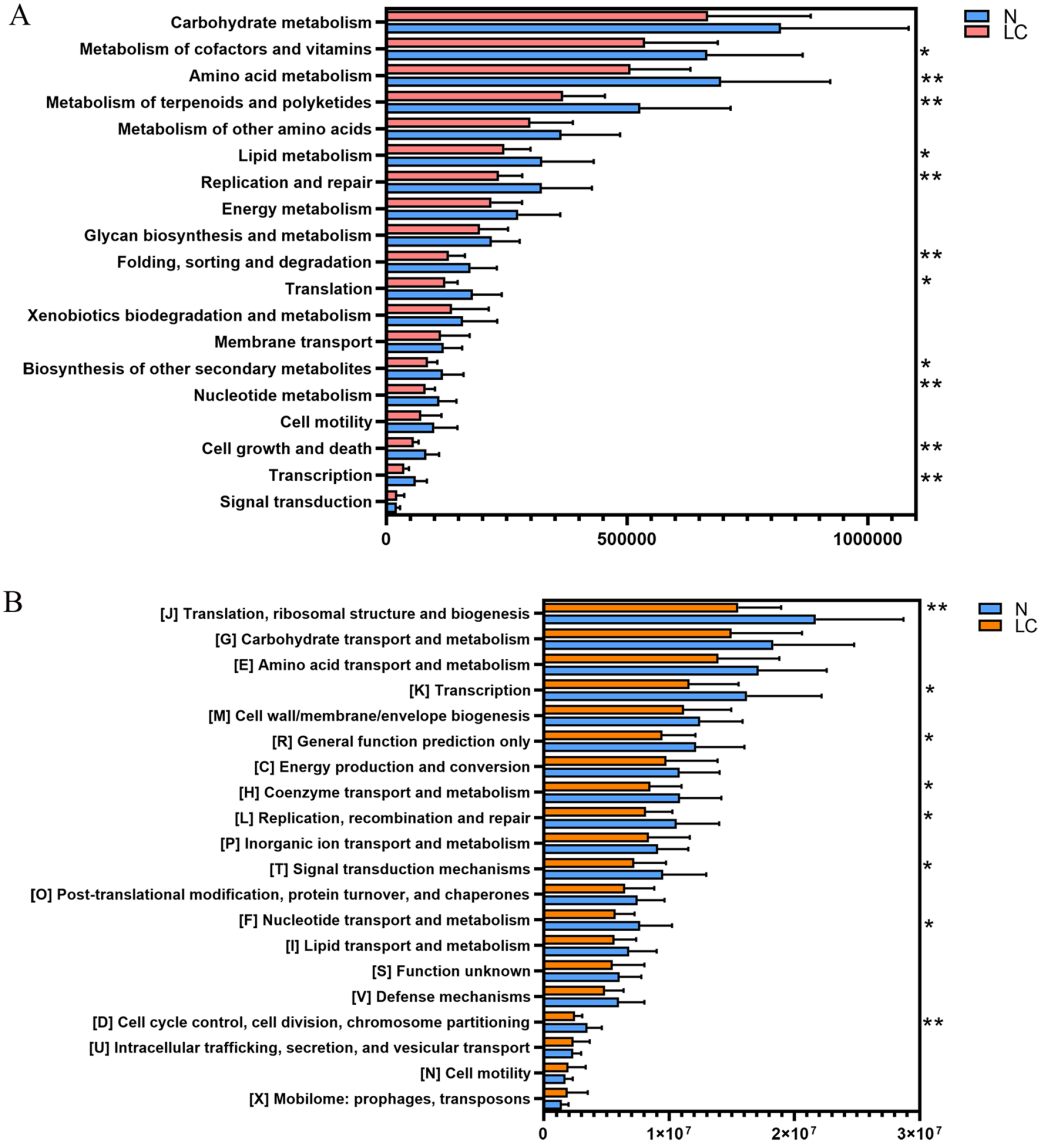
Comparison of KEGG pathway outcomes (**A**) and COG categories data (**B**) between the LC and N groups. *p < 0.05, **p < 0.01, LC group compared with N group.

The KEGG pathways and COG annotation were compared to explore the potential differences in the functional composition of the microbiome between patients with liver carcinoma and the control group. The functional intestinal microbiome composition of patients with hepatocellular carcinoma differed from that of the control group. KEGG pathway analysis shows a decline in the Metabolism of cofactors and vitamins, Amino acid metabolism, Metabolism of terpenoids and polyketides, Replication and repair, Translation, Biosynthesis of other secondary metabolites, Nucleotide metabolism, Cell growth and death, and Transcription (Fig. 6A). Additionally, COG analysis shows that Transcription, General function prediction only, Coenzyme transport and metabolism, Replication, recombination and repair, Signal transduction mechanisms, Nucleotide transport and metabolism, Cell cycle control, cell division, chromosome partitioning in liver cancer patients are significantly lower than those in the healthy control group (p < 0.05) (Fig. 6B).

### Correlations between gut microbiota and clinical, biochemical parameters

**Figure 7:**
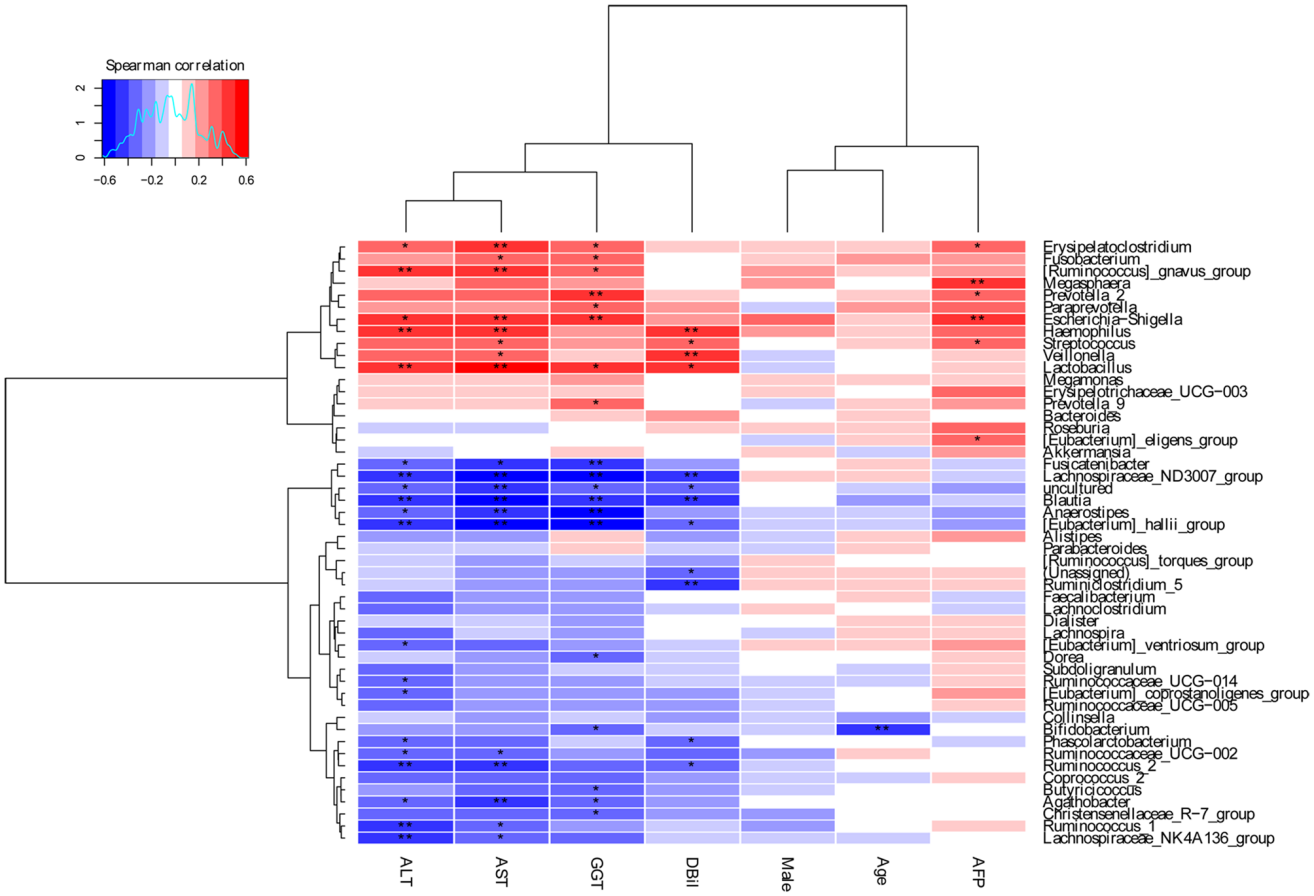
Spearman's correlations between different dominant genera and clinical, biochemical traits. *p < 0.05, **p < 0.01.

The Spearman correlations of the gut microbiota at the genus level and variables are described in Fig. 7. Age is negatively associated with the abundance of *Bifidobacterium* (p < 0.01). *Lachnospiraceae_ ND3007_ group*, *Blautia*, *[Eubacterium]_hallii_group, Fuscatenibacter* and *Anaerostipes* are negatively associated with ALT, AST and GGT levels (p < 0.05), respectively. *Erysipelatoclostridium*, *[Ruminococcus]_gnavus_group*, *Escherichia-Shigella* and *Veillonela* are positively correlated with ALT, AST and GGT levels (p < 0.05), respectively. Alpha-fetoprotein (AFP) is positively associated with the abundance of *Erysipelatoclostridium*, *Magasphaera*, *Prevotella 2*, *Escherichia-Shigella*, *Streptococcus* and *[Eubacterium]_eligens_group* (p < 0.05), respectively.

### Gut microbiota-based prediction of HCC

**Figure 8:**
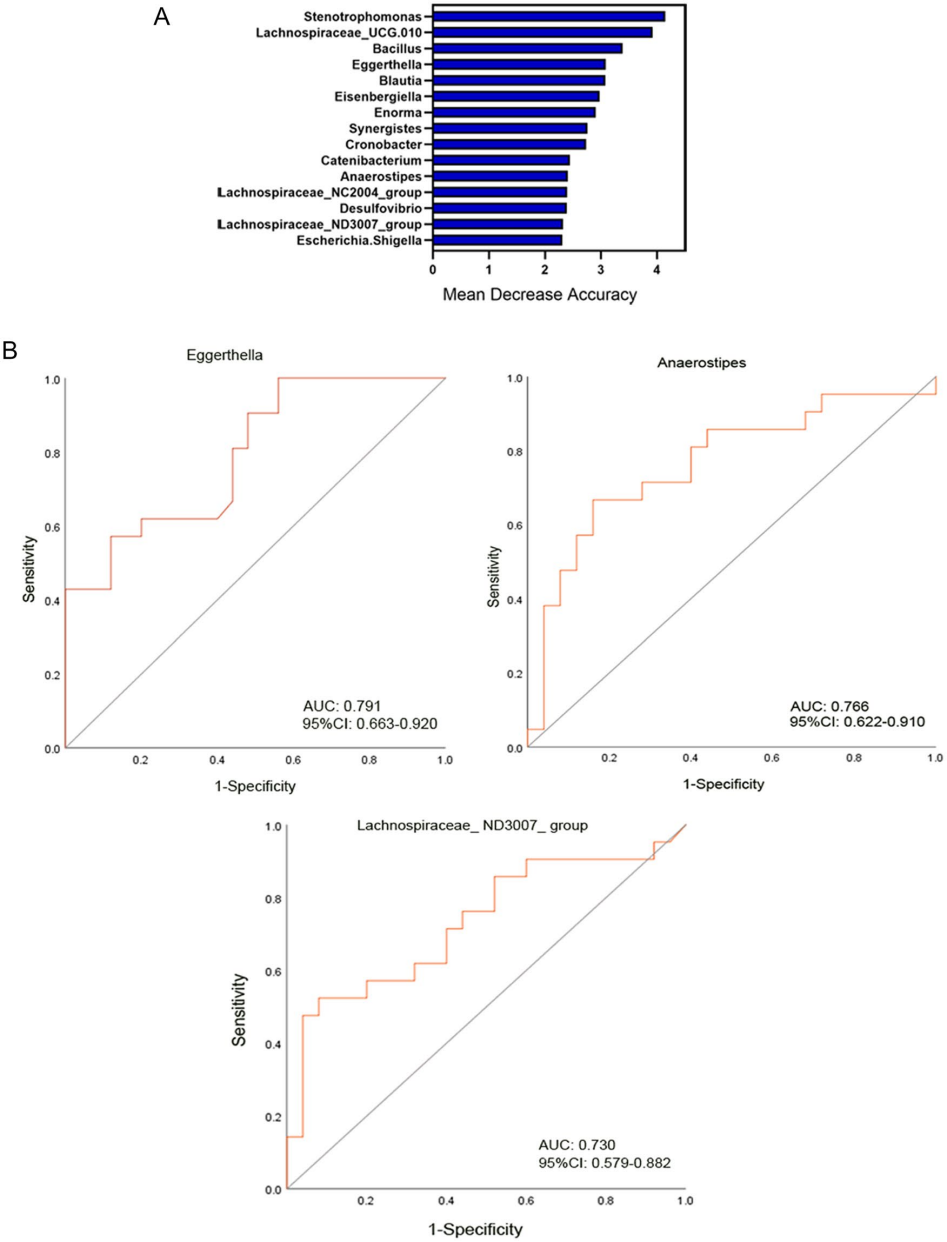
Gut microbiota–based prediction of HCC. (**A**) Identification of gestational diabetes mellitus (GDM) markers by random forest models; (**B**) Receiver operating characteristic (ROC) curves of genera-based diagnostic biomarkers for HCC.

Finally, random forest models were used to assess the ability of the genus abundance profiles to predict the diagnosis of HCC (Fig. 8A). Three genera afforded optimal HCC detection: *Eggerthella*, *Anaerostipes* and *Lachnospiraceae_ ND3007_ group*. The areas under the receiver operating characteristic curves (AUCs under the ROCs) are 0.791, 0.766 and 0.730, respectively (Fig. 8B).

## Discussion

Recently, the relationship between gut microbiota and human health has been attracting more and more attention^20–23^. Complete intestinal mucosal barrier and hepato-intestinal circulation are important conditions for maintaining gut microbiota homeostasis in the host, and microbiota-related metabolites and body immunity may be the primary mechanism of the interaction between gut microbiota and liver^24^. Some studies have highlighted that the gut microbiota of liver cancer patients has changed significantly compared with the healthy subject regarding microbiota diversity, structure, and quantity^25^.

The age range of hepatocellular carcinoma patients is substantial, and the gut microbiota varies extensively over such a large age range. Hepatocellular carcinoma is a cancer, with 80% of cases diagnosed among patients 70 years or older^26^. Mounting studies distinguish the treatment of cancers between young and elderly people^27,28^. Therefore, this study focuses on gut microbiota differences in elderly patients with hepatocellular carcinoma and is the first known gut microbiome study of elderly patients with hepatocellular carcinoma.

The apparent differences in the two groups of samples regarding α diversity or β diversity are reported in this study, indicating that gut microbiota characteristics in patients with hepatocellular carcinoma have changed significantly. The data suggest that changing gut microbiota characteristics correlates specifically with liver cancer. At the genus level, compared with the normal group, the abundance of *Blautia, Fusicatenibacter, Anaerostipes, Lachnospiraceae_ND3007_group, CAG-56, Eggerthella, Lachnospiraceae_FCS020_group and Olsenella* in liver cancer group are decreased in the hepatocellular carcinoma group. Ren's study^25^ reported that, compared with the normal control, the abundance of butyric acid-producing bacteria such as *Ruminococcus*, *Oscillibacter*, *Faecalibacterium*, *Clostridium IV,* and *Coprococcus* decreased in the early HCC group, which differs from the current study. In the Chen^29^ study, *Blautia, Eubacterium hallii group,* and *Ruminococcus gnavus group* were significantly lower in HCC patients, and *Proteobacteria* were positively correlated with age, male numbers, and creatine. In the Deng^30^ study, the HCC group had the highest *Collinsella* and lowest *Lachnospiraceae* among the three groups. In the Liu^31^ study, *Megamonas, Lachnospira, Eubacterium ventriosum* and *Lachnospiraceae_UCG-001* were significantly decreased in non-HBV non-HCV related-HCC patient samples compared with the healthy control sample. In the Ponziani^32^ study, the HCC microbiome was significantly higher in the abundance of *Enterococcus, Ruminococcus, Bacteroides, Phascolarctobacterium and Oscillospira* genera.

In this study, *Eggerthella* significantly decreases in the liver cancer group*.* No studies on the association between *Eggerthella* and liver cancer have been identified. Harris^33^ reported that *Eggerthella.* lenta DSM 2243 and strain C592 participate in bile acid oxidation, prompting speculation that bile acid oxidation identified in liver carcinoma patients could be associated with *Eggerthella.*

No previous reports have been found investigating the association between *Lachnospiraceae_ND3007_group* and *Olsenella* and liver cancer, and the mechanism remains unknown. Species from the *Lachnospiraceae ND3007*^34^*, Lachnospiraceae_FCS020_groups*^35^ and *Olsenella* ^36^ have been reported as SCFA-producing bacteria. Butyrate impacts gut physiology and the immune system and is associated with Treg cell differentiation activation in the intestine through histone acetylation^37^. One possibility is that decreasing SCFA abundance might be an important cause or correlation of carcinogenesis.

One study showed that *CAG-56* decreased in the gut microbiota of HIV-infected patients^38^, suggesting a potential interplay between HIV-related microbiota, immune dysfunction, and comorbid metabolic conditions. The current study shows that the abundance of *CAG-56* decreases in LC patients. No previous reports were found on the association between *CAG-56* and liver cancer, leading to the speculation that *CAG-56* is related to the patient's immune system.

The current data indicate that the abundances of *Escherichia-Shigella, Prevotella_2, Tyzzerella_4, Cronobacter and Erysipelatoclostridium* are significantly increased in the LC group. *Escherichia-Shigella* is known as a lipopolysaccharide-producing bacteria. In Ren's study ^25^, the abundance of lipopolysaccharide-producing bacteria such as *Klebsiella* and *Haemophilus* increased. Reportedly, pathogenic gram-negative bacteria families belonging to the *Proteobacteria* phylum, such as *Enterobacteriaceae* comprising *Shigella, Escherichia coli, Klebsiella, Proteus,* and *Enterobacter* are increased in HCC, NAFLD, HBV, and cirrhosis^39^. *Enterobacteriaceae* are ethanol-producing bacteria capable of causing liver damage and have been correlated with serum tumor necrosis factor (TNF)-α, interleukin-1(IL-1), and IL-6 levels^40,41^. In the Tang study, pro-inflammatory bacteria (*Veillonella*, *Escherichia-shigella*) are increased in the liver cirrhosis group^42^. Yin^43^ reports that the abundance of colorectal cancer-promoting bacteria such as *Escherichia/Shigella* and *Enterococcus* was evidently elevated in post-*Fusobacterium nucleatum* treatment. *Cronobacter* can cause severe infections in restricted populations, leading to death or chronic sequelae as a foodborne pathogen^44^. In this study, *Cronobacter* is significantly increased in the liver carcinoma group, an association that has not previously been reported. The pathways and mechanisms that link this correlation need to be further explored.

The KEGG pathways and COG annotation were compared to explore the potential differences in the functional composition of the microbiome between elderly patients with hepatocellular carcinoma and the control group. The KEGG pathways and COG annotation analysis showed that the dysbiosis of gut bacteria in primary liver carcinoma is associated with several pathways, including Amino acid metabolism, Replication and repair, Nucleotide metabolism, Cell motility, Cell growth and death, and Transcription. These KEGG pathways and COG annotation alterations facilitated decreased function in liver cancer patients. *Firmicutes* play an essential role in the process that could transform undigested carbohydrates and proteins into acetic acid, a valuable energy source for organisms^45^. Thus, *Firmicutes* deficiencies lead to a decreased function of biosynthetic and metabolic processes. In Liu's study, HCC patients showed fewer amino acid and glucose metabolism pathways. Yu et al*.* have indicated a potential correlation between gut microbiota and thyroid carcinoma, suggesting that the microbial changes observed in thyroid carcinoma patients cause a decline in aminoacyl-tRNA biosynthesis, homologous recombination, mismatch repair, DNA replication, and nucleotide excision repair through functional prediction^46^.

Spearman's correlation analysis was undertaken to identify the relationships between bacterial genera and blood biomarkers in elderly patients with HCC. Age is negatively associated with the abundance of *Bifidobacterium*. *Lachnospiraceae_ ND3007_ group*, *Blautia*, [*Eubacterium]_hallii_group*, *Fuscatenibacter* and *Anaerostipes* negatively correlate with ALT, AST and GGT levels. AFP is positively associated with the abundance of *Erysipelatoclostridium*, *Magasphaera*, *Prevotella 2*, *Escherichia-Shigella*, *Streptococcus* and *[Eubacterium]_eligens_group*. In Huo's study^47^, *Anaerostipes*, *Fusicatenibacter*, *Bifidobacterium* and *Faecalibacterium* were negatively associated with AFP, ALT, AST, and PIVKA. In contrast, *Lactobacillus* and *Klebsiella* were positively associated with AFP, ALT, AST, and PIVKA, which differs slightly from the current study.

The data from this study support the hypothesis that gut microbiota contains non-invasive markers of HCC. Thus, fivefold cross-validation using a Random Forest model was undertaken. The AUCs under the ROCs of the *Eggerthella*, *Anaerostipes* and *Lachnospiraceae_ ND3007_ group* genera were 0.791, 0.766, and 0.730, respectively. These microbiotic markers have not previously been proposed as biomarkers to predict the development or presence of HCC.

However, this study has some limitations. The study's main limitation is the small sample size, so future studies including more patients are warranted to verify our findings. Additionally, because of the use of 16S rRNA data, associations at species and functional profiles of gut microbiome composition could not be explored. Metagenomics and metabonomics approaches could be applied to explore the in-depth mechanism.

In conclusion, the data for the first known gut microbiome study in elderly patients with hepatocellular carcinoma are presented. Potentially, specific microbiota can be used as a characteristic index for screening, diagnosis, and prognosis of gut microbiota changes in elderly patients with hepatocellular carcinoma and even as a therapeutic clinical target. A more detailed investigation of the relationship between the gut microbiome and hepatocellular carcinoma is warranted.

## Data Availability

Illumina sequencing reads were uploaded to the SRA under accession number PRJNA905530. Any other data supporting this study's conclusions are available from the corresponding author upon request.
